# Arsenic trioxide exerts synergistic effects with cisplatin on non-small cell lung cancer cells via apoptosis induction

**DOI:** 10.1186/1756-9966-28-110

**Published:** 2009-08-08

**Authors:** Hecheng Li, XiaoLi Zhu, Yawei Zhang, Jiaqing Xiang, Haiquan Chen

**Affiliations:** 1 Department of Thoracic Surgery, Fudan University Cancer Hospital/Cancer Institute, Shanghai, PR China; 2 Department of Oncology, Fudan University Shanghai Medical College, Shanghai 200032, PR China; 3 Department of Pathology, Fudan University Cancer Hospital/Cancer Institute, Shanghai, PR China

## Abstract

**Background:**

Despite multidisciplinary treatment, lung cancer remains a highly lethal disease due to poor response to chemotherapy. The identification of therapeutic agents with synergistic effects with traditional drugs is an alternative for lung cancer therapy. In this study, the synergistic effects of arsenic trioxide (As_2_O_3_) with cisplatin (DDP) on A549 and H460 non-small cell lung cancer (NSCLC) cells were explored.

**Methods:**

A549 and H460 human lung cancer cells were treated with As_2_O_3 _and/or DDP. Cell growth curves, cell proliferation, cell cycle, and apoptosis of human cancer cell lines were determined by the 3-(4,5)-dimethylthiahiazo (-z-y1)-3,5-di-phenytetrazoliumromide (MTT) method, clonogenic assay, and flow cytometry (FCM). Apoptosis was further assessed by TUNEL staining. Cell cycle and apoptosis related protein p21, cyclin D1, Bcl-2, bax, clusterin, and caspase-3 were detected by western blot.

**Results:**

MTT and clonogenic assay showed As_2_O_3 _within 10^-2 ^μM to 10 μM exerted inhibition on the proliferation of NSCLC cells, and 2.5 μM As_2_O_3 _exerted synergistic inhibition on proliferation with 3 μg/ml DDP. The combination indices (CI) for A549 and H460 were 0.5 and 0.6, respectively, as confirmed by the synergism of As_2_O_3 _with DDP. FCM showed As_2_O_3 _did not affect the cell cycle. The G0/G1 fraction ranged from 57% to 62% for controlled A549 cells and cells treated with As_2_O_3 _and/or DDP. The G0/G1 fraction ranged from 37% to 42% for controlled H460 cells and cells treated with As_2_O_3 _and/or DDP. FCM and TUNEL staining illustrated that the combination of As_2_O_3 _and DDP provoked synergistic effects on apoptosis induction based on the analysis of the apoptosis index. Western blotting revealed that the expression of cell cycle related protein p21 and cyclin D1 were not affected by the treatments, whereas apoptosis related protein bax, Bcl-2, and clusterin were significantly regulated by As_2_O_3 _and/or DDP treatments compared with controls. The expression of caspase-3 in cells treated with the combination of As_2_O_3 _and DDP did not differ from that in cells treated with a single agent.

**Conclusion:**

As_2_O_3 _exerted synergistic effects with DDP on NSCLC cells, and the synergistic effects were partly due to the induction of caspase-independent apoptosis.

## Background

Lung cancer is the number one cause of cancer mortality in both males and females worldwide [1]. Despite multidisciplinary treatment, lung cancer is still a highly lethal disease due to late detection and resistance to chemotherapy. The identification of new therapeutic agents that exert synergistic effects in combination with traditional cytotoxic agents is an alternative strategy for the systemic treatment of lung cancer.

Recent evidence indicates that arsenic trioxide (As_2_O_3_) may induce clinical remission in patients with acute promyelocytic leukemia (APL), and several investigations show that As_2_O_3 _induced programmed cell death in APL cell lines [2-5]. DDP, a platinum-containing anticancer drug, is one of the most commonly used cytotoxic agents for the treatment of lung cancer. Due to the poor therapeutic effects of current cytotoxic-agents on lung cancer, the ability of As_2_O_3 _to induce apoptosis in non-small cell lung cancer cells was explored in the present study, and the synergistic effects of As_2_O_3 _with DDP on A549 and H460 lung cancer cells were analyzed.

## Methods

### Cell culture and reagents

Human lung cancer A549 and H460 cell lines were obtained from the ATCC and maintained in RPMI 1640 medium with 10% fetal bovine serum and 1% penicillin. As_2_O_3 _was purchased from Yida Pharmaceutical Co.(GMP, Ha'erbin, PR. China) and DDP was from Bristol-Myers Squibb Co.(Shanghai, PR. China).

### MTT assay

Briefly, cells were seeded at a density of 2,000 to 5,000 cells/well in 96-well plates and incubated overnight. After treatment with As_2_O_3_, DDP, or their combination (described below), 3-(4, 5-methylthiazol-2-yl)-2, 5-diphenyl-tetrazolium bromide (MTT) was added (50 μL/well) for 4 hours. Solubilization of the converted purple formazan dye was accomplished by placing cells in 100 μL of 0.01 N HCl/10% SDS and incubating them overnight at 37°C. The reaction product was quantified by absorbance at 570 nm. All samples were repeated three times, and data were analyzed by Student's t test.

### In vitro clonogenic assay

Human lung carcinoma cells were counted after trypsinization. Cells were serially diluted to appropriate concentrations and removed into 25-cm^2 ^flasks in 5-mL medium in triplicate per data point. After various treatments, cells were maintained for 8 days. Cells were then fixed for 15 minutes with a 3:1 ratio of methanol:acetic acid and stained for 15 minutes with 0.5% crystal violet (Sigma) in methanol. After staining, colonies were counted by the naked eye, with a cutoff of 50 viable cells. Error bars represent ± SE by pooling of the results of three independent experiments. Surviving fraction was calculated as (mean colony counts)/(cells inoculated)*(plating efficiency), where plating efficiency was defined as mean colony counts/cells inoculated for untreated controls.

### Cell cycle and apoptosis analysis

Flow cytometry analysis of DNA content was performed to assess the cell cycle phase distribution as described previously[6]. Cells were harvested and stained for DNA content using propidium iodide fluorescence. The computer program Multicycle from Phoenix Flow System (San Diego, CA, USA) was used to generate histograms which were used to determine the cell cycle phase distribution and apoptosis. TUNEL staining was also used to detect apoptosis as described previously [7]. The TUNEL stained apoptotic cells were separately numbered in four randomly selected microscopic fields (400*) and graphed.

### Western blot

After various treatments, cells were washed with ice-cold PBS twice before the addition of lysis buffer (20 mM Tris, 150 mM NaCl, 1 mM EDTA, 1% Triton X-100, 2.5 mM sodium NaPPi, 1 mM phenylmethylsulfonyl fluoride, and leupeptin). Protein concentrations were quantified separately by the Bio-Rad Bradford assay. Equal amounts of protein were loaded into each well and separated by 10% SDS PAGE, followed by transfer onto nitrocellulose membranes. Membranes were blocked using 5% nonfat dry milk in PBS for 1 hour at room temperature. The blots were then incubated with anti-p21, anti-cyclin D1, anti-bax, anti-bcl-2, anti-clusterin, and anti-caspase-3 antibodies (Santa Cruz Biotechnology, Santa Cruz, CA) at 4°C overnight. Blots were then incubated in secondary antibody conjugated with HRP (1:1000; Santa Cruz Biotechnology) for 1 hour at room temperature.

Immunoblots were developed using the enhanced chemiluminescence (ECL) detection system (Amersham, Piscataway, NJ) according to the manufacturer's protocol and autoradiography.

## Results

### As_2_O_3 _exerted synergistic effects with DDP on the proliferation of A549 and H460

**Figure 1:**
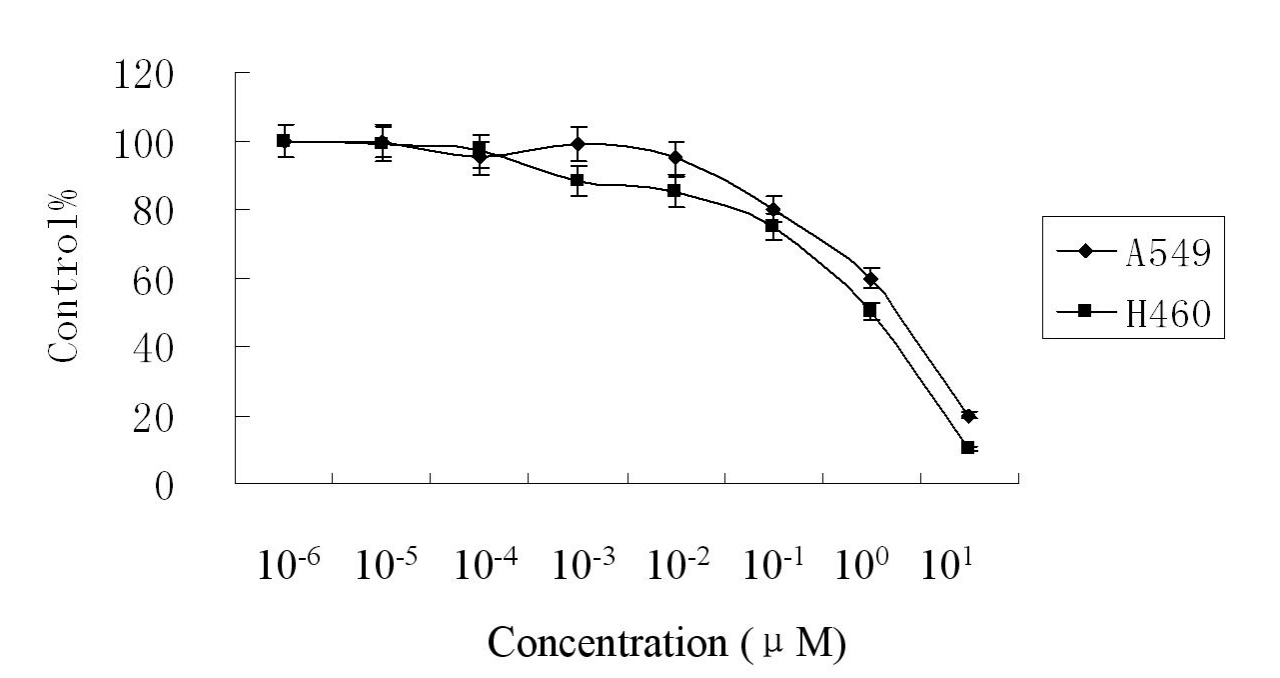
**Dose response curves for effects of As_2_O_3 _on A549 and H460 lung cancer cell proliferation**. Cells were treated with different concentrations of As_2_O_3 _(10^-6^–10 μM) for 72 hours. Proliferation was analyzed by MTT assay. As_2_O_3 _concentrations of 10^-2 ^μM to 10 μM inhibited A549 cell proliferation at 72 hours.

**Figure 2:**
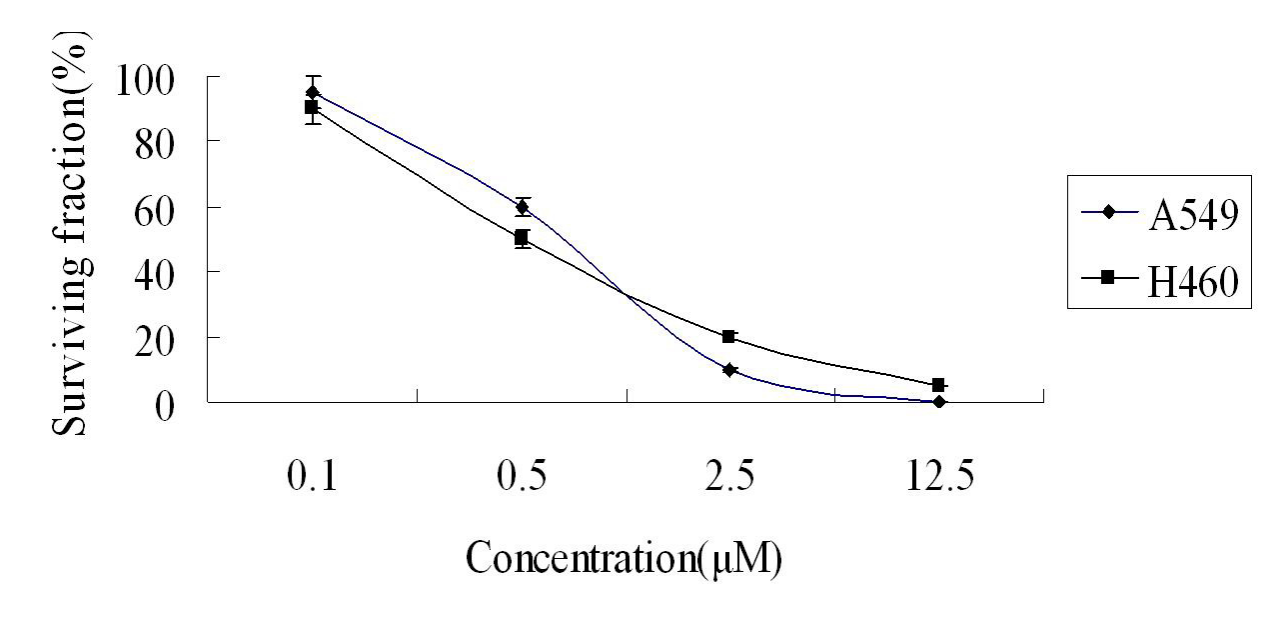
**Clonogenic assay of the effects of As_2_O_3 _on the proliferation of A549 and H460 cells**. *In vitro *clonogenic assays showed that 10^-1 ^μM to 12.5 μM As_2_O_3 _inhibited the proliferation of A549 and H460 cells. Surviving fraction was calculated as (mean colony counts)/(cells inoculated) × (plating efficiency), where plating efficiency was defined as mean colony counts/cells inoculated for untreated controls.

**Figure 3:**
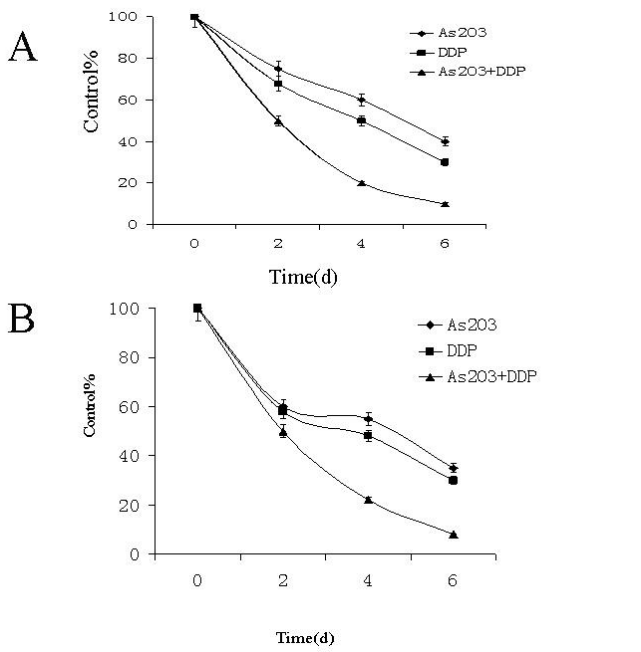
**Synergistic effects of As_2_O_3 _and DDP in lung cancer cell lines**. A. The synergistic effect of As_2_O_3 _and DDP in the treatment of A549 cells. MTT assay results showed that 2.5 μM As_2_O_3 _and 3 μg/ml DDP exerted synergistic inhibition effects on A549 cells at 48 hours. B. The synergistic effect of As_2_O_3 _and DDP in the treatment of H460 cells. MTT assay results showed that 2.5 μM As_2_O_3 _and 3 μg/ml DDP exerted synergistic inhibition effects on H460 cells at 48 hours.

**Figure 4:**
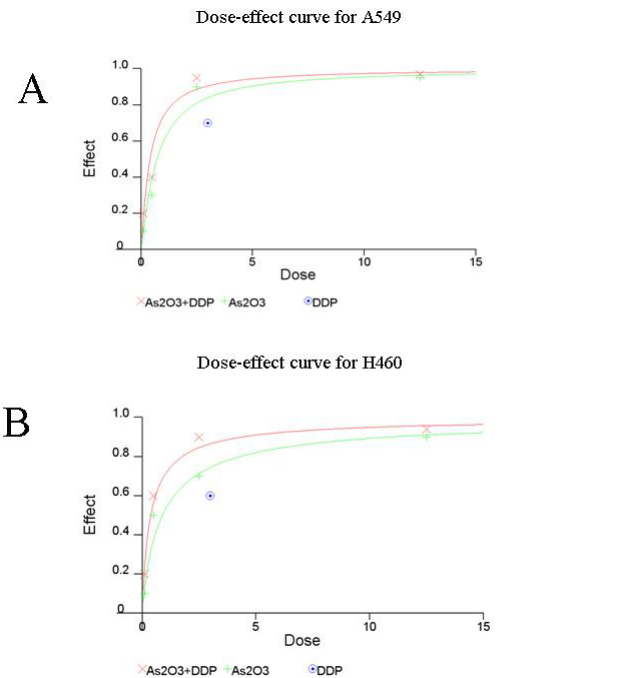
**Dose effect curve for A549 (A) and H460 (B) cells**. The concentration of DDP was 3 μg/ml and the concentration for As_2_O_3 _ranged from 0.1 μM to 12.5 μM. CalcuSyn™ (Version 2.0, Biosoft, Inc., UK) was used for dose-effect curves and to determine the combination indices (CI).

The MTT assay showed that 10^-2 ^μM to 10 μM inhibited the proliferation of A549 and H460 at 72 hours (Fig. 1). In vitro clonogenic assay proved 10^-1 ^μM to 12.5 μM As_2_O_3 _inhibited the proliferation of A549 and H460 cells (Fig. 2). MTT assay results showed that 2.5 μM As_2_O_3 _and 3 μg/ml DDP exerted synergistic inhibition effects on A549 and H460 cells at 48 hours. (Fig. 3A,B). To confirm the synergistic effects of As_2_O_3 _with DDP CalcuSyn™ program (Version 2.0, Biosoft, Inc., UK) was explored to make dose-effect curves and to determine the combination indices (CI) (Fig. 4A,B). The CI for A549 and H460 were 0.5 and 0.6, respectively which confirmed the synergism of As_2_O_3 _with DDP.

### As_2_O_3 _did not significantly affect the cell cycles of A549 and H460 cells

**Figure 5:**
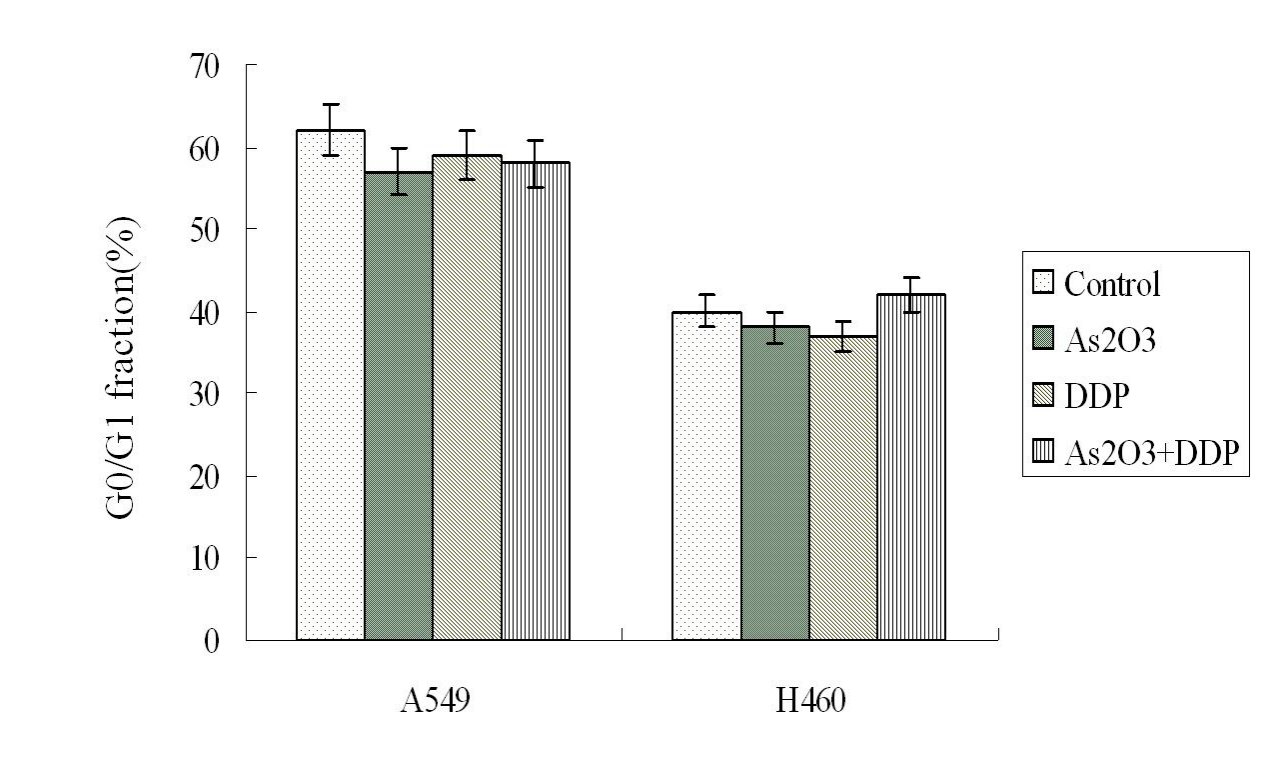
**G0/G1 fraction analysis**. FCM cell cycle analysis showed that the treatment of As_2_O_3 _and/or DDP did not significantly affect G0/G1 fractions of A549 and H460 cells compared with those of the control. The G0/G1 fraction ranged from 57% to 62% for control A549 cells and for A549 cells treated with As_2_O_3 _and/or DDP, and from 37% to 42% for control H460 cells and for H460 cells treated with As_2_O_3 _and/or DDP.

A549 cells were treated with 2.5 μM As_2_O_3 _and/or 3 μg/ml DDP for 48 hours. FCM cell cycle analysis showed that the treatment of As_2_O_3 _and/or DDP did not significantly alter G0/G1 fractions of A549 cells compared with those of the control. The G0/G1 fraction ranged from 57% to 62% for controlled A549 cells and cells treated with As_2_O_3 _and/or DDP; the G0/G1 fraction ranged from 37% to 42% for controlled H460 cells and cells treated with As_2_O_3 _and/or DDP (Fig. 5). Western blot analysis showed that As_2_O_3 _and/or DDP did not affect the expression of cell cycle related protein p21 and cyclin D1 (data not shown).

### As_2_O_3 _and/or DDP induced apoptosis of A549 and H460 cells

**Figure 6:**
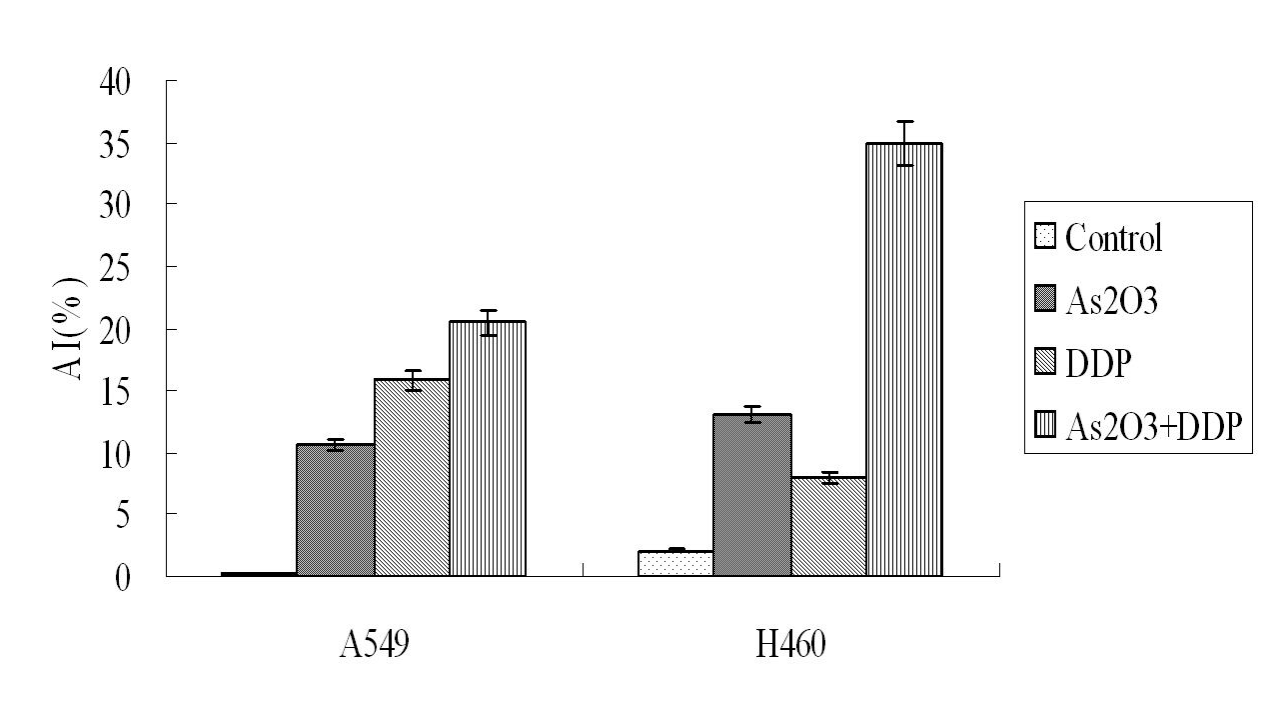
**FCM cell cycle analysis of apoptotic index (AI) for cells treated with As_2_O_3 _and/or DDP**. AI for the control A549 cells and cells treated with As_2_O_3_, DDP, or the combination were 0.25 ± 0.01%, 10.6 ± 0.53%, 15.85 ± 0.79%, and 20 ± 1%, respectively; the AI for the control H460 cells and cells treated with As_2_O_3_, DDP, or the combination were 1.95 ± 0.11%, 13.6 ± 0.65%, 7.53 ± 0.43%, and 35.6 ± 1.71%, respectively.

**Figure 7:**
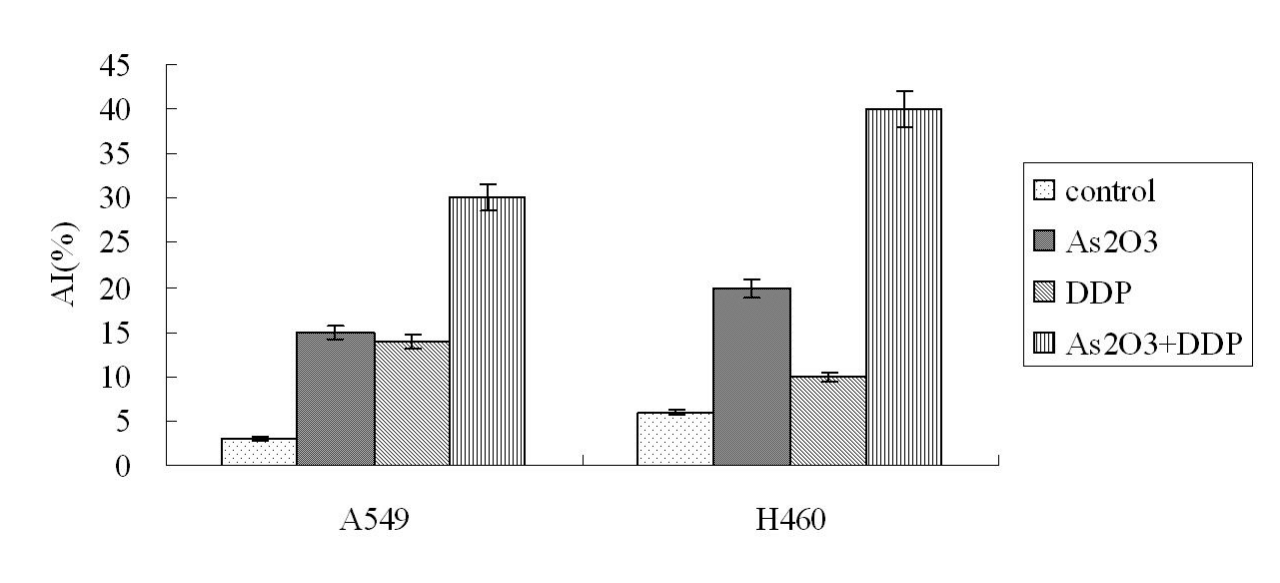
**TUNEL staining analysis**. With TUNEL staining, the AI for the control A549 cells and cells treated with As_2_O_3_, DDP, or the combination were 3.1 ± 0.16%, 15.41 ± 0.77%, 14 ± 0.7%, and 30 ± 1.5%, respectively; the AI for the control H460 cells and cells treated with As_2_O_3_, DDP, or the combination were 5.95 ± 0.25%, 18.6 ± 1.13%, 9.53 ± 0.49%, and 40.6 ± 2.11%, respectively.

**Figure 8:**
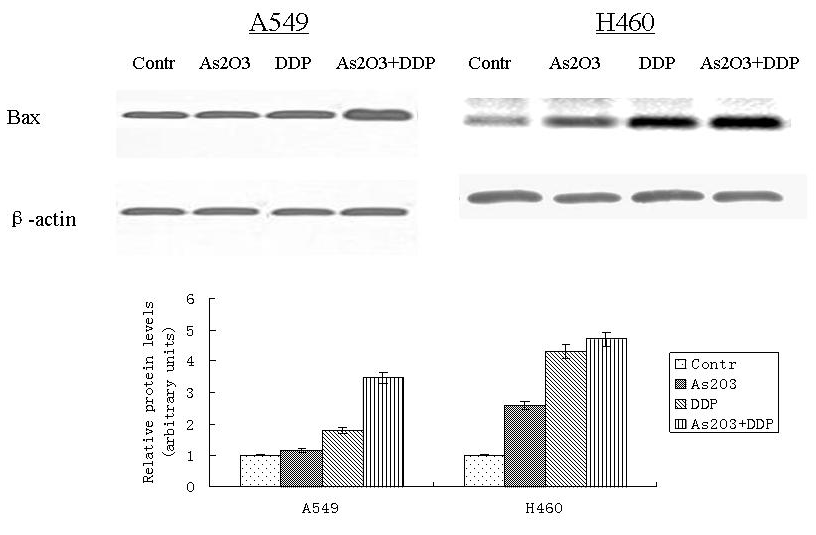
**Western blot analysis of Bax expression in lung cancer cell after different treatments**. Bax expression was 2-fold greater in A549 cells treated with As_2_O_3 _and DDP than in control cells. Bax expression was 3.7-fold greater in H460 cells treated with As_2_O_3 _and DDP than in control cells.

**Figure 9:**
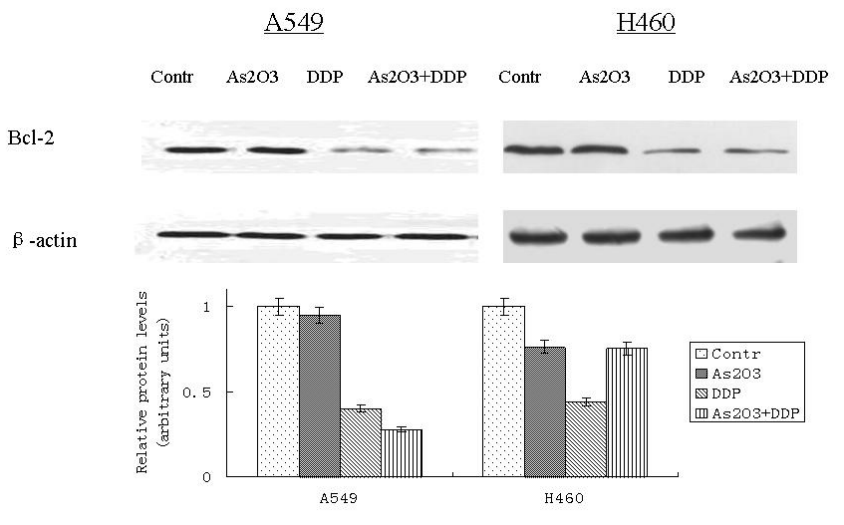
**Western blot analysis of Bcl-2 expression in lung cancer cells after different treatments**. Bcl-2 expression was 72% less in As_2_O_3 _and DDP-treated A549 cells than in controls, and it 25% less in As_2_O_3 _and DDP-treated H460 cells than in controls.

**Figure 10:**
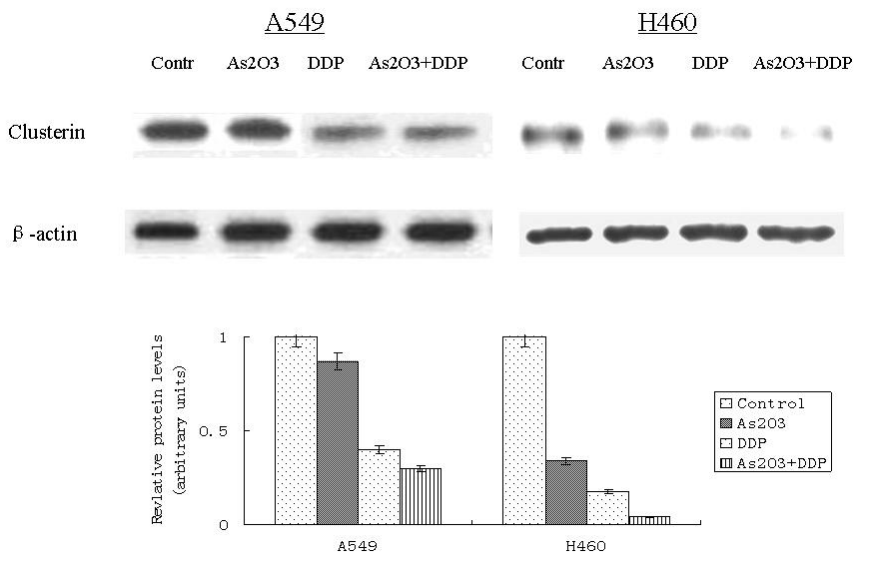
**Western blot analysis of clusterin expression in lung cancer cells after different treatments**. Clusterin expression was 70% less in As_2_O_3 _and DDP-treated A549 cells than in controls, and in H460 cells, clusterin expession was 90% less with treatment of the combination of As_2_O_3 _and DDP than in controls.

**Figure 11:**
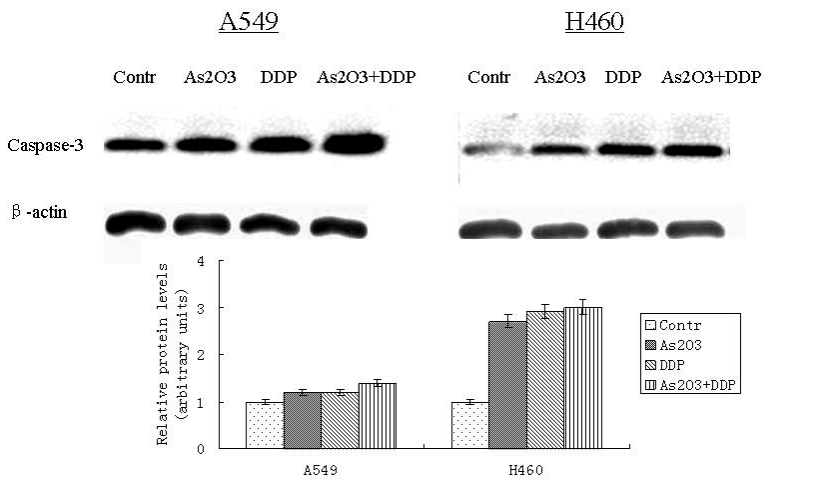
**Western blot analysis of caspase-3 expression in lung cancer cells after different treatments**. For both A549 and H460 cells, caspase-3 expression increased with treatment of As_2_O_3 _and/or DDP, but caspase-3 expression did not differ in cells treated with the combination of As_2_O_3 _and DDP and cells treated with each single agent.

A549 cells were treated with 2.5 μM As_2_O_3 _and/or 3 μg/ml DDP for 48 hours. FCM analysis showed the apoptotic indices (AI) for the controlled A549 cells and cells treated with As_2_O_3_, DDP, or the combination were 0.25 ± 0.01%, 10.6 ± 0.53%, 15.85 ± 0.79%, and 20 ± 1%, respectively. The AI for the controlled H460 cells and cells treated with As_2_O_3_, DDP, or the combination were 1.95 ± 0.11%, 13.6 ± 0.65%, 7.53 ± 0.43%, and 35.6 ± 1.71%, respectively (Fig. 6). As_2_O_3 _and DDP significantly increased the AI compared with the control cells. TUNEL staining was performed to further confirm AI results from FCM analysis. With TUNEL staining, the AI for the control A549 cells, cells treated with As_2_O_3_, DDP, or the combination were 3.1 ± 0.16%, 15.41 ± 0.77%, 14 ± 0.7%, and 30 ± 1.5%, respectively. The AI for the control H460 cells, cells treated with As_2_O_3_, DDP, or the combination were 5.95 ± 0.25%, 18.6 ± 1.13%, 9.53 ± 0.49%, and 40.6 ± 2.11%, respectively (Fig. 7). Western blot analysis showed Bax expression increasing by 2-fold in the A549 cells treated with As_2_O_3 _and DDP over levels in control cells. In H460 cells treated with As_2_O_3 _and DDP, Bax expression was 3.7 times greater than in the control (Fig. 8). Bcl-2 expression was 72% less in the As_2_O_3 _and DDP treated A549 cells than in control cells, and 25% less in the As_2_O_3 _and DDP treated H460 cells than in control cells (Fig. 9). Expression of another tumor suppressed protein, clusterin, was 70% less in the As_2_O_3 _and DDP treated A549 cells than in control cells, and in H460 cells, clusterin expression was 90% less with treatment with the combination of As_2_O_3 _and DDP than in control cells (Fig. 10). For both A549 and H460, caspase-3 expression increased with the treatment of As_2_O_3 _and/or DDP over control levels, but caspase-3 expression was not different in cells treated with the combination of As_2_O_3 _and DDP and cells treated with each single agent (Fig. 11).

## Discussion and conclusion

Our *in vitro *study showed that As_2_O_3 _is an effective reagent that inhibits the proliferation of A549 and H460 lung cancer cells. As_2_O_3 _cytotoxicity was due to the induction of apoptosis but not cell cycle arrest. FCM and TUNEL assay analyses showed that As_2_O_3 _significantly induced apoptosis. When As_2_O_3 _and DDP were combined, a synergistic effect was found in the treatment of A549 and H460 cells. Protein assays showed that the combination of As_2_O_3 _and DDP affected apoptosis-related proteins such as Bcl-2, Bax, and clusterin but not caspase-3, while the use of each single agent did not. The changes in apoptosis-related protein expression partly contributed to the effect of As_2_O_3 _on lung cancer cells.

Since lung cancer is a lethal disease due to late detection and resistance to chemotherapy, this study was conducted to determine whether As_2_O_3 _could exert synergistic effects in combination with traditional cytotoxic-agents on lung cancer cell death. Although As_2_O_3 _has been an effective treatment for the acute promyelocytic leukemia, the mechanism by which As_2_O_3 _induces cell death remains poorly understood. Recent reports suggest that As_2_O_3 _causes DNA damage, oxidative stress, and mitochondrial dysfunction [8,9]. In addition, As_2_O_3 _treatment results in cell-cycle arrest in MCF-7 HeLa cells [10]; however, our results demonstrate that cell cycle is not significantly affected by As_2_O_3_, since the G1/0 fraction and cell cycle-related protein expression did not change significantly with As_2_O_3 _treatment. The inconsistency between these findings may be due to different mechanisms of action by As_2_O_3 _in various cell lines. Our results were consistent with previous studies that indicated that proapoptotic Bcl-2 family members, Bcl-2 and Bax, are involved in the apoptosis of cancer cells induced by As_2_O_3 _[11,12]. Previous studies show that clusterin is a caspase-independent apoptosis-related protein and it is a potential target in the treatment of non-small cell lung cancer [13-15]. Here, we showed that the synergistic effects of As_2_O_3 _and DDP might be due, in part, to clusterin-mediated apoptosis. Depending on the cell system investigated, As_2_O_3_-induced cell death has been associated with caspase-dependent apoptosis, as well as caspase-independent death pathways [16-18]. In this study, the combination of As_2_O_3 _and DDP increased caspase-3 expression, which indicates that caspase might be involved in apoptosis induced by As_2_O_3 _or DDP. However, the combination of As_2_O_3 _and DDP did not affect caspase-3 expression compared with cells treated with a single agent, which suggests that the synergistic effects are more likely to be caspase-independent. This study showed caspase-independent death pathways that involved Bcl-2, Bax, and clusterin were the primary mechanism by which As_2_O_3 _exerts synergistic effects with DDP on NSCLC cells.

In conclusion, As_2_O_3 _exerted synergistic effects with DDP on lung cancer cells. The proliferation inhibition might be partly due to the induction of apoptosis. Based on our study, As_2_O_3 _may be a promising agent in the treatment of lung cancer, although further *in vitro *and *in vivo *studies are necessary to elucidate the mechanism by which As_2_O_3 _induces apoptosis.

## Competing interests

The authors declare that they have no competing interests.

## Authors' contributions

As principle investigator HL and HC had full access to all of the data in this study and take responsibility for the accuracy of the data analysis. Study concept and design: HL, XZ and JX. MTT, Clonogenic assay, Flow cytometry assay, TUNEL assay and western blot: XZ, HL. Analysis and interpretation of data: XZ, HL. Drafting of the manuscript: HL, XZ. Critical revision of the manuscript: JX, HC. Supervision: YZ, JX and HC.
